# Supplementary material for: Inhibitory effects of polysaccharides from Korean ginseng berries on LPS-induced RAW264.7 macrophages
Source: PLoS One. 2023 Nov 28;18(11):e0294675. doi: 10.1371/journal.pone.0294675 (PMC10684074; doi:10.1371/journal.pone.0294675)

Original western blot gel image data

Inhibitory effects of polysaccharides from Korean ginseng berries on LPS-induced RAW264.7 macrophages

Replication 1

- **p-NF-κB-p65**

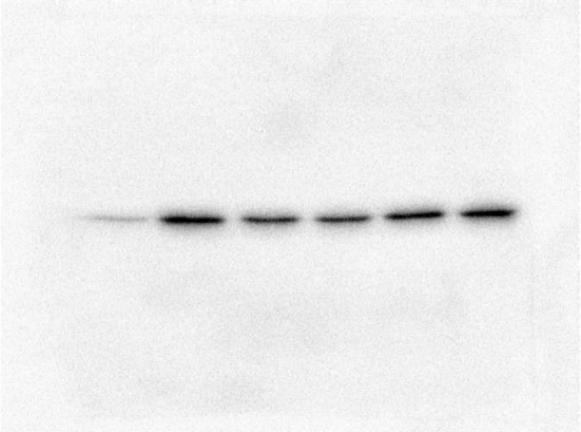

- **p-p38**

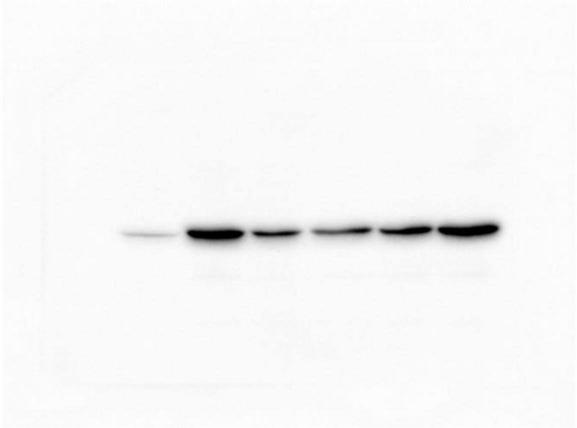

- **p-JNK**

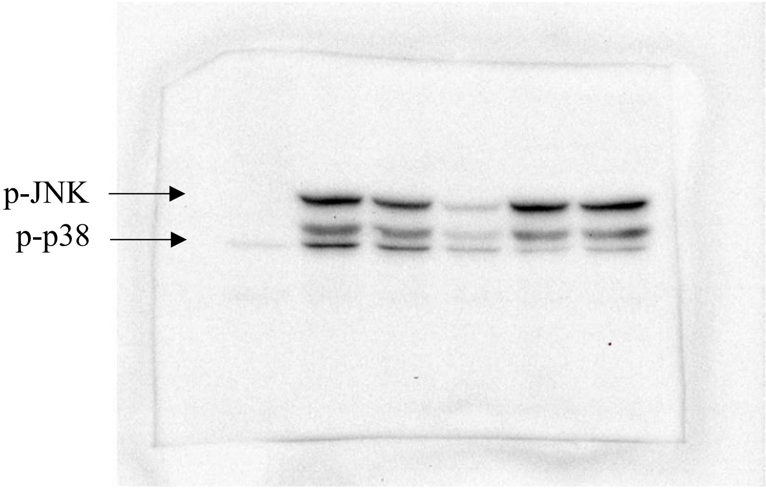

- **p-ERK 1/2**

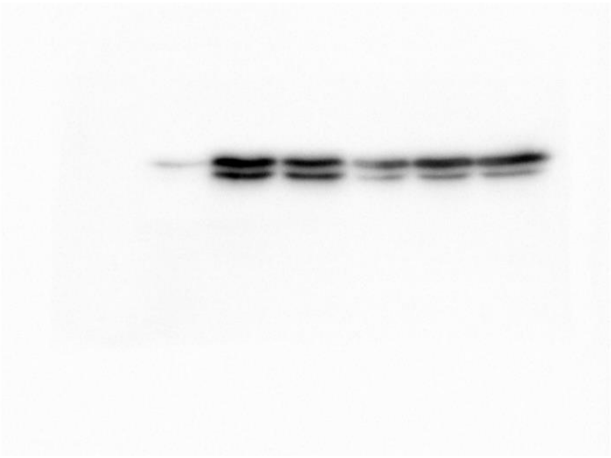

- **α-tubulin**

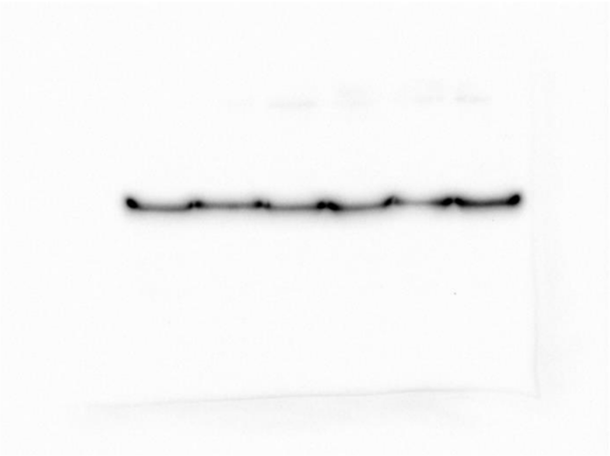

Replication 2

- **p-NF-κB-p65**

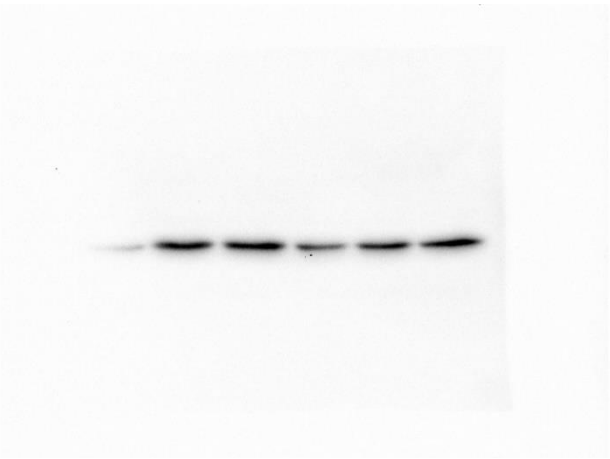

- **p-p38**

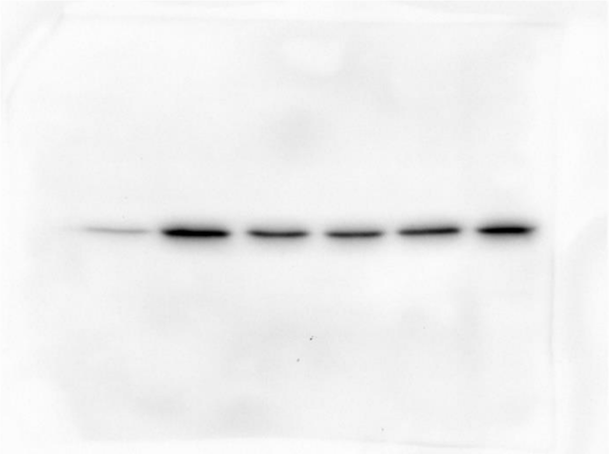

- **p-JNK**

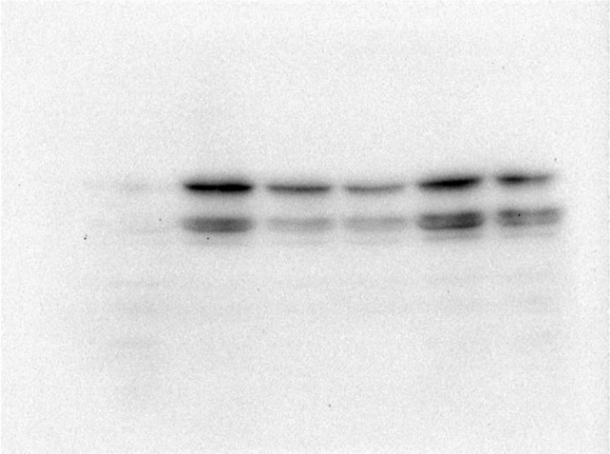

- **p-ERK 1/2**

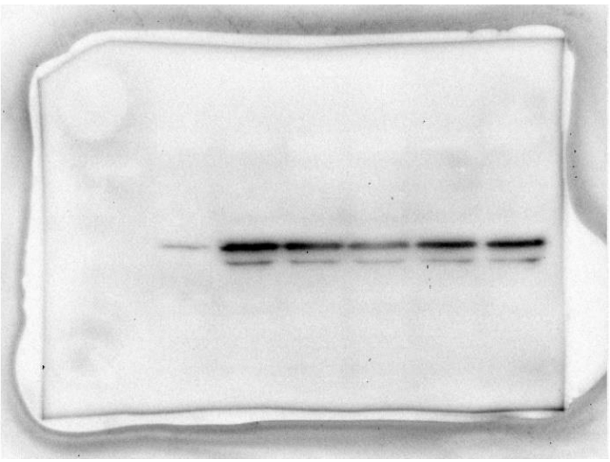

- **α-tubulin**

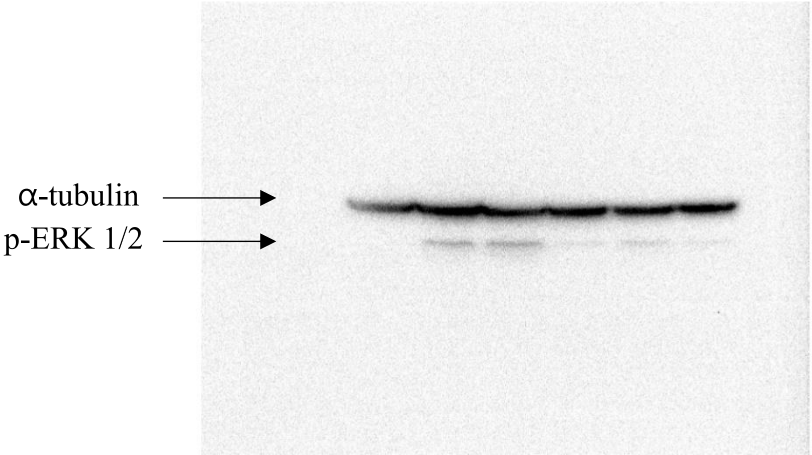

Replication 3

- **p-NF-κB-p65**

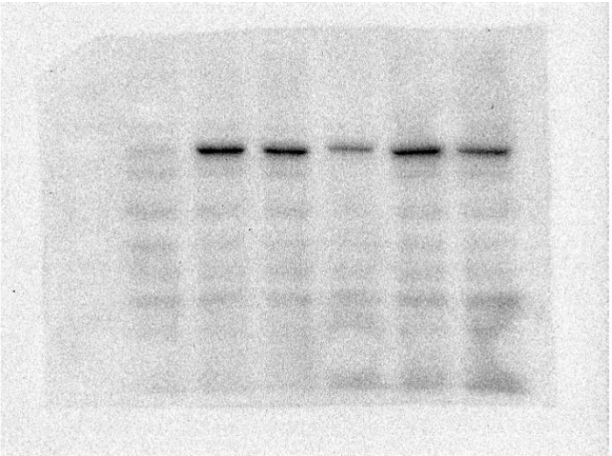

- **p-p38**

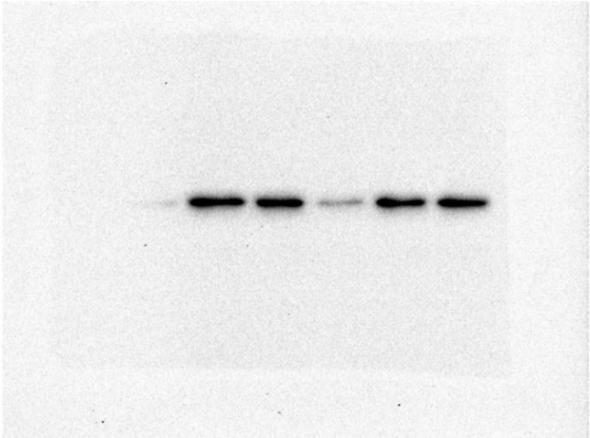

- **p-JNK**

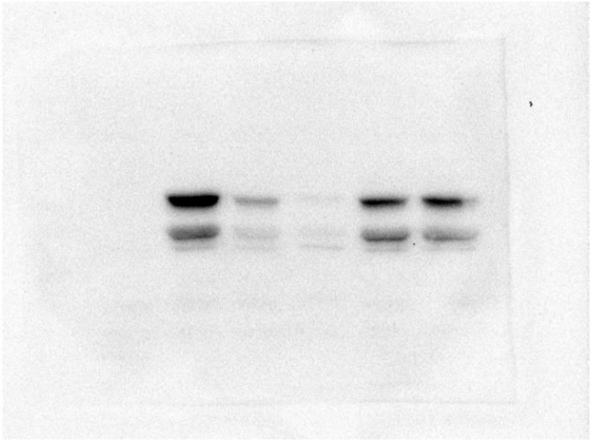

- **p-ERK 1/2**

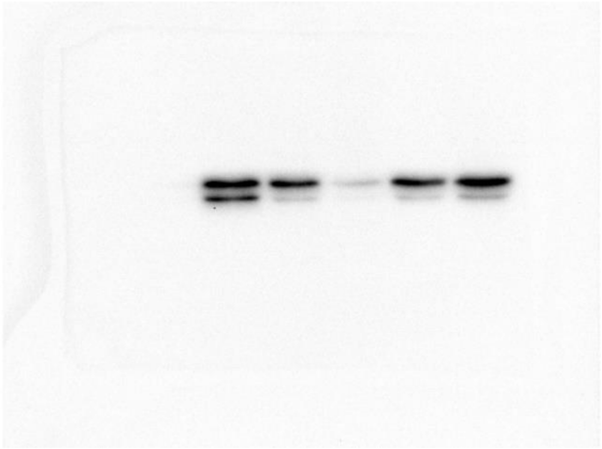

- **α-tubulin**

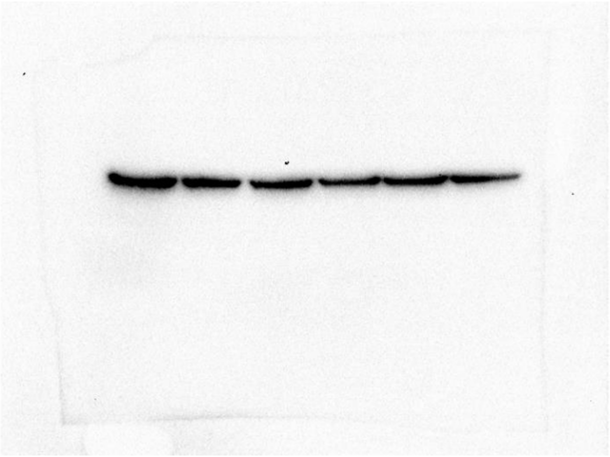

Supplement: S1 Fig — (PDF) [file pone.0294675.s001.pdf]
